# The Design of Novel 3D-Printed, Moulded, and Oral Viscous Budesonide Formulations for Paediatrics: A Comparative Evaluation of Their Mucoadhesive Properties

**DOI:** 10.3390/pharmaceutics16101338

**Published:** 2024-10-18

**Authors:** María Magariños-Triviño, Eduardo Díaz-Torres, Javier Suárez-González, Ana Santoveña-Estévez, José B. Fariña

**Affiliations:** 1 Departamento de Ingeniería Química y Tecnología Farmacéutica, Campus de Anchieta, Universidad de La Laguna, 38200 La Laguna, Spain; ediaztor@ull.edu.es (E.D.-T.); jsuarezg@ull.edu.es (J.S.-G.); ansanto@ull.edu.es (A.S.-E.);; 2 Instituto Universitario de Enfermedades Tropicales y Salud Pública de Canarias, Universidad de La Laguna (ULL), Avenida Astrofísico Francisco Sánchez, s/n., 38200 La Laguna, Spain; 3 Programa de Doctorado en Ciencias Médicas y Farmacéuticas, Desarrollo y Calidad de Vida, Universidad de La Laguna, 38200 La Laguna, Spain

**Keywords:** 3D printing, individualised medicines, paediatric, eosinophilic oesophagitis, budesonide, orodispersible, viscous formulations, printlet, moulded tablets, semi-solid extrusion

## Abstract

Background/Objectives: Paediatric eosinophilic oesophagitis (EoE) treatment is challenging due to the limited number of age-appropriate formulations. This study aims to develop and evaluate oral viscous suspensions and solid formulations of budesonide (BUD), focusing on their in vitro mucoadhesive properties, to enhance drug delivery and therapeutic outcomes in paediatric EoE. Methods: This study encompasses the development of oral viscous suspensions and orodispersible solid formulations (moulded tablets and 3D-printed dosage forms) containing BUD. The formulations underwent quality control tests as per the European Pharmacopoeia, chemical stability assessments, and an in vitro evaluation of their mucoadhesiveness properties. Results: A validated analytical method enabled accurate BUD quantification and efficient extraction, and all developed formulations demonstrated chemical stability for 30 days, meeting Ph. Eur. quality standards. Three-dimensional printing using SSE successfully produced 1 mg and 0.5 mg BUD printlets, complying with quality tests for conventional tablets. Formulations containing xanthan gum (L2-XG and P1-0.5-XG) exhibited superior mucoadhesive properties. L2-XG showed significantly higher mucoadhesion than L1-MC. Among the solid formulations, P1-0.5-XG demonstrated the highest mucoadhesive properties. Conclusions: This is the first study to develop solid oral dosage forms of BUD at a very low dose, specifically for paediatric use. The results highlight the potential of 3D printing for developing individualised orodispersible BUD formulations with improved bioadhesion for paediatric EoE treatment. The L2-XG formulation and the XG-containing printlets are the most promising formulations in terms of increasing contact time with the oesophageal mucosa, which could translate into improved therapeutic efficacy in this patient population.

## 1. Introduction

Eosinophilic oesophagitis (EoE) is a chronic, immune-mediated inflammatory disorder characterised by eosinophil infiltration of the oesophageal mucosa. This infiltration triggers a cascade of disorders, including tissue remodelling and fibrosis, leading to oesophageal dysfunction [1,2].

Current therapeutic strategies for EoE include medication, dietary modifications, and oesophageal dilation for the management of complications [3]. Only a limited number of treatments have gained approval for EoE, such as budesonide (BUD) effervescent tablets for adult use in most European countries (Jorveza^®^) and dupilumab for patients aged 12 and above [4,5]. Jorveza^®^, developed primarily for adult patients, offers a distinctive approach to EoE management. However, its safety and efficacy in paediatric populations remain unestablished [6]. Consequently, medications commonly employed in paediatric clinical practice, such as proton pump inhibitors (PPIs) and swallowed topical corticosteroids (STCs) like oral viscous budesonide (OVB) and fluticasone propionate (FP), are often prescribed off-label for EoE management. Both medications have been shown to be safe and effective in inducing and maintaining clinical, endoscopic, and histological remission in patients [1,7]. STCs provide the highest clinical–histological response rates (66%) compared to PPI (44%) and diet (42%) [8]. Within STCs, budesonide demonstrates a superior histological remission rate compared to fluticasone, of 76% and 68%, respectively [9].

Furthermore, recent research underscores the variability in practice patterns regarding the use of topical corticosteroids in paediatric EoE. Syverson et al., in 2023, found that OVB is favoured in younger children (<5 years), while FP is more common in adolescents (13–18 years) [10]. Reinforcing this observation, a multicentre European study in 2024 highlighted BUD orodispersible tablets as the most effective STC, while also emphasising the considerable variability in STC utilisation across clinical practice [11]. The induction dose for paediatric eosinophilic oesophagitis is 0.5 mg BUD twice daily for children <10 years and 1 mg twice daily for those ≥10 years [12].

Most of the prior research on BUD formulations for EoE has centred on oral viscous suspensions, often utilising gums, and particularly xanthan gum (XG), as the vehicle, with the goal of developing standardised preparations that can prolong drug contact with the oesophageal mucosa and improve therapeutic outcomes. XG had a greater mucosal contact time compared to sucralose or other gums like guar gum. These investigations have shown that the penetration of BUD into the mucosa is influenced by several factors, including the molecule’s characteristics and the formulation’s mucoadhesive properties and viscosity. While increased viscosity can enhance drug contact time and tissue penetration by reducing outflow, excessive viscosity may hinder syringe extrusion, posing a practical challenge in drug administration [3,13,14]. In vitro mucoadhesiveness studies have shown that oral viscous BUD formulations maintain contact with the oesophageal mucosa for approximately 30 min [3], consistent with the recommended fasting period after Jorveza^®^ administration [6]. Clinical studies often utilise nuclear scintigraphy to assess the oesophageal emptying rate and estimate the contact time of BUD formulations with the oesophageal mucosa, expressed as the area under the curve (AUC) generated from scintigraphy images. A higher AUC, indicating prolonged contact time of the drug with the oesophageal mucosa, has been shown to correlate with a more significant reduction in eosinophil count and improved clinical outcomes in patients with EoE [15,16,17].

Budesonide exists as a mixture of two epimers, 22R and 22S, due to the presence of asymmetric acetyl groups in its chemical structure [18,19]. Both epimers exhibit biological activity, but the 22R epimer demonstrates greater potency compared to the 22S epimer [18]. Nevertheless, both epimers have similar terminal half-lives and undergo extensive first-pass metabolism in the liver, resulting in low systemic bioavailability and minimised systemic effects. The presence of these two epimers contributes to budesonide’s favourable properties, including its high affinity for corticosteroid receptors and enhanced topical potency [19,20].

Considering the extensive variability within paediatric populations, a systematic approach to paediatric formulation development is crucial to ensure safe and effective treatment [21]. The ability to precisely tailor dosage forms to individual patient needs is paramount in this population group, where factors such as age, swallowing ability, and potential allergies or intolerances can significantly impact treatment success [22,23]. This study encompasses a comprehensive evaluation of diverse BUD formulations, including oral viscous suspensions and solid orodispersible dosage forms (moulded tablets and 3D-printed dosage forms), to address these multifaceted challenges. Oral viscous suspensions are particularly relevant for patients who are unable to or have difficulty swallowing solid dosage forms. Solid orodispersible forms, considered the most suitable dosage forms by the World Health Organization (WHO) [24], offer advantages in palatability and swallowability. They also exhibit appropriate stability profiles without the need for the preservatives commonly included in liquid formulations [25].

Among these solid orodispersible forms, we have focused on moulded tablets, a well-established and readily accessible approach in pharmaceutical compounding. This technique involves melding moistened powder blends with a binder solution to create orodispersible tablets [26,27]. This simple preparation process facilitates their implementation in pharmacy services. On the other hand, 3D-printed dosage forms (printlets) created via semi-solid extrusion (SSE) provide the unique advantage of complete customization; the API concentration can be fully modified by adjusting the print settings and geometry, enabling the production of dosage forms with lower doses and adaptable sizes, thus creating a final pharmaceutical form on demand and according to individual patient needs [15,17,18,19].

The objective of this study is to develop and evaluate novel, non-commercially available, oral, liquid viscous and solid formulations of budesonide for paediatric EoE. This involves evaluating the quality control, stability tests, and in vitro mucoadhesive properties of oral viscous BUD formulations, developing new mucoadhesive orodispersible solid formulations containing 1 and 0.5 mg of BUD using moulding and 3D printing by semi-solid extrusion techniques, and, finally, comparing the effectiveness of these liquid and solid formulations in terms of their in vitro mucoadhesiveness.

## 2. Materials and Methods

### 2.1. Materials

Budesonide (BUD), edetate disodium (EDTA), citric acid monohydrate, glycerine, methylcellulose-1000, nipagin sodium (methylparahydroxibenzoate), nipasol (propyl parahydroxybenzoate), saccharin sodium, sodium benzoate, sodium citrate, lactose monohydrate, ethanol, apple flavouring essence, and xanthan gum were supplied by Acofarma (Barcelona, Cataluña, Spain). Croscarmellose sodium (Ac-Di-Sol^®^) was provided by FMC Corporation (Philadelphia, PA, USA). Purified water was obtained from a Puranity TU 12 water purification system (VWR, Radnor, PA, USA).

### 2.2. Quality Target Product Profile

First, the quality target product profile (QTTP) of the formulations to be developed was established, outlining the necessary attributes to ensure their final quality, efficacy, and safety [28,29]. The corresponding QTPP is presented in Table 1.

### 2.3. Composition of the Formulations

The composition of each formulation, including the percentage of each component, was determined based on prior laboratory testing [26] and is summarised in Table 2 and Table 3. The oral viscous formulation based on methylcellulose (L1-MC) was proposed by our research group [30], while the second composition based on xanthan gum (L2-XG) was included based on a recommendation from the Spanish Society of Hospital Pharmacy [31]. The solid formulations (M1, P1, P0.5, P0.5-XG) were developed in response to a specific demand from a paediatric hospital unit. The target BUD dose for each orodispersible dosage form was 1 mg. Additionally, 0.5 mg BUD printlets were also developed.

### 2.4. Preparation of Formulations

#### 2.4.1. Preparation of Oral Viscous Suspensions

The following procedure was employed for the preparation of both oral viscous liquid formulations (L1-MC and L2-XG). Purified water was added to a 250 mL beaker. All excipients, except for methylcellulose (for L1-MC) or xanthan gum (for L2-XG), were slowly added under continuous stirring until complete dissolution. Subsequently, methylcellulose or xanthan gum was gradually sprinkled onto the surface of the water, which was stirred gently to prevent lump formation, until a homogenous viscous dispersion was obtained. BUD was pulverised in a mortar, moistened with a pre-weighed amount of glycerine, and then incorporated into the viscous dispersion. The final formulation was transferred to a 125 mL amber PET bottle with a dispensing cap suitable for syringes.

#### 2.4.2. Preparation of Moulded Tablets

API and excipients were weighed, milled, and thoroughly mixed with a mortar and pestle to prepare moulded tablets using the Optima Tablet^®^ system (Farmalabor, Canosa di Puglia, Italy) [27]. Employing the wet granulation technique, 1.7 mL of binder solution (PVP 5% *w*/*w*, 30% ethanol, 2% apple essence, and 63% purified water) was incorporated to attain the intended consistency, akin to that of wet sand. Subsequently, the wet mass was gently spread onto a cavity plate using a stainless steel flexible-blade spatula. Consequently, the peg plate was raised through the cavity plate, resulting in the formation of tablets atop the pins [26]. The tablets were then allowed to dry for 6 h at room temperature (25.1 ± 0.1 °C/64.0 ± 0.6% RH). Two batches comprising a total of 100 orodispersible moulded tablets were prepared for this study.

#### 2.4.3. Preparation of Printlets

- Preparation of semi-solid masses

API and excipients were milled separately using a mortar and pestle. Each component was then added to an Unguator^®^ mixer jar in the following order: BUD, lactose monohydrate, Ac-Di-Sol, and xanthan gum in the case of P0.5-XG. Then, 8 mL of binder solution (5% PVP, 30% ethanol, 2% apple essence, and 63% purified water) was gradually added, 2 mL at a time, mixing for 15 s at 650 rpm at each interval, to achieve a wet mass with a homogeneous texture suitable for extrusion [26,32]. The wet mass was transferred to a 20 mL syringe with a Luer lock (B. Braun Medical Inc. OEM, Melsungen, Germany) and Fisnar QuantX™ 20 ga Pink nozzles (Fisnar, Glasgow, UK) and set in a pharmaceutical 3D printing platform (M3DIMAKER, FabRx Ltd., London, UK) equipped with a pressure-instrumentalized SSE motor-driven printhead (Laguna SSE printhead, FabRx Ltd., London, UK).

- Extrudability analysis of the semi-solid masses

To assess potential variations in rheological behaviour, an extrudability analysis was conducted to characterize the physical properties of each of the semi-solid masses developed before printing using the SSE-P sensor integrated into the printing platform. Extrusion pressure, yield point (the pressure needed to initiate the extrusion), and steady flow parameters, critical for determining paste extrudability and flowability, and ultimately printlet quality, were evaluated. The Texturometer Utility for M3DIMAKER Studio (FabRx Ltd., UK), integrated into the 3D printing platform, was utilised to establish the compression/shrinkage cycle; see each step in Table 4.

Throughout the cycle, the accumulated weight of extruded semi-solid material was recorded using a precision scale ENTIRIS153I-1S (d = 0.001 g) (Sartorius, Göttingen, Germany) positioned on the 3D printing building plate.

This characterisation allowed for the determination of the semi-solid paste’s density from its extruded mass (mg) as a function of plunger displacement (mm) and for the determination of the dimensions of printed objects required to deliver the target dose of BUD (P1: 1 mg BUD, P0.5 and P0.5-XG: 0.5 mg BUD) [32,33]. P3Diatrics software version 1.3. facilitated the calculation of the final object dimensions.

- Printing Settings and Quality Control of the Printing process

The printlets were designed in Autodesk Fusion 360 (version 2.0.9011, Autodesk Inc., San Francisco, CA, USA) and were exported as .stl files for processing in the 3D printing software Slic3r. The printlets were crafted as a torus shape with a 6.22 mm radius, 2.4 mm height, and 10% infill. Printing utilised polypropylene as the surface material, with a speed of 10 mm/s, a first-layer thickness of 0.4 mm, and thickness of the subsequent layers of 0.6 mm. Following printing, the printlets were stored on the printing platform at room temperature (25.2 ± 0.1 °C/64.4 ± 0.6% RH) for 6 h to dry.

To ensure that the process was controlled throughout the production of the printlets and to identify any obstructions or the presence of air that could have an impact on the dosage forms’ final quality attributes (CQAs), the applied pressure values of the SSE Laguna printhead were collected using the built-in data logger of the M3DIMAKER 3D printing platform [34].

### 2.5. UPLC Method Validation

BUD content was analysed by reversed-phase Ultra-High-Performance Liquid Chromatography (UHPLC) in an Acquity UPLC^®^ H-Class System (Waters Corporation, Milford, MA, USA), using Astra 6.0.1 as the acquisition software (Chromatographic Manager, Waters Corporation, Milford, MA, USA).

The UHPLC method was adapted and validated from a previously published method [35]. The chromatographic conditions were an Acquity^TM^ Premier Peptide BEH C18 (50 mm × 2.1 mm id, 1.7 µm) reversed-phase column; acetonitrile: phosphate buffer 23 mM adjusted to pH 3.2, with orthophosphoric acid as the mobile phase at a proportion of 32:68 (*v*/*v*); flow rate of 0.7 mL/min; wavelength of 254 nm, 10 μL injection volume; and an analysis time of 3.5 min. All chemicals and reagents were of analytical grade. All samples and solvents were filtered with 0.2 μm pore-sized polyester filters (Chromafil ^®^ Xtra PET-20/25 Disposable syringe filter, Millipore, Billerica, MA, USA) before proceeding with chromatographic analysis. To calibrate the system and monitor performance, a sample solution containing the BUD was analysed daily.

The validation of the analytical method was conducted according to the ICH guideline, using standard solutions with concentrations ranging from 2 to 16 μg/mL [36]. An analysis of variance (ANOVA) was carried out to confirm the linearity of the method.

The method’s precision (repeatability) was determined by a six-fold analysis of the same sample. System accuracy was expressed as percentage recovery by assay of a known drug quantity (n = 6). The detection and quantification limits, based on the standard deviation of the response and slope, were also checked.

In addition, to ensure that the method can follow the degradation of the BUD being analysed with UV, a standard sample was subjected to a basic medium (0.1 M NaOH).

### 2.6. Extraction Methods

The quantification method must be capable of analysing the content and obtaining the API amount declared in each dosage form from a complex matrix (mainly non-soluble excipients), ensuring that the excipients do not interfere with the chromatographic analysis and that the sample treatment is appropriate for the quantitative analysis of BUD in the formulations. For this purpose, 1 mg of BUD and the right amount of each excipient for the liquid or solid formulations were weighed and the following detailed extraction method was used for liquids or solids. The procedure was repeated ten times for each type of formulation, and the average amount was calculated and expressed as the labelled content.

#### 2.6.1. Extraction for Liquid Formulations (L1-MC, L2-XG)

For this purpose, the right amounts of BUD and glycerine, according to the composition of each type of liquid formulation, L1-MC or L2-XG (Table 2), were weighed and transferred to a 5 mL flask; the volume was completed with 1% methylcellulose gel (L1-MC) or 2% xanthan gum gel (L2-XG) and vortexed to ensure proper sample homogenization. All of the contents were transferred to a 25 mL flask, and absolute ethanol was added to approximately 75% of the volume. The mixture was placed in an ultrasonic bath for 20 min to facilitate the dissolution of BUD. The volume was completed with ethanol, and it was transferred to a conical tube to be centrifuged for 25 min at 25 °C and 4500 rpm to sediment the gel. After centrifugation, an aliquot of the supernatant sufficient was diluted with mobile phase to be analysed in the UHPLC system.

#### 2.6.2. Extraction for Solid Formulations (M1, P1, P0.5, P0.5-XG)

For this purpose, the right amounts of BUD and each excipient, according to the composition of each type of moulded tablet (M1) or printlet (P1, P0.5, P0.5-XG), were weighed and dissolved in 12.5 mL of water. Then, 10 mL of absolute ethanol was added and the solution was placed in an ultrasonic bath for 10 min to ensure the BUD was completely dissolved. In the case of printlets, the samples were filtered using 110 mm filter paper (Albet LabScience, Barcelona, Spain). Finally, both were diluted with mobile phase to be analysed in the UHPLC system.

### 2.7. Quality Control

#### 2.7.1. Quality Control for Liquid Formulations

The appearance, organoleptic properties, and pH of the liquid formulations were evaluated according to the Spanish National Formulary [37]. The pH of each formulation was measured in a Crison GLP 21 pH Meter. A 5 mL sample was taken from each solution at 0 and 30 days and the measurements were conducted in triplicate at 25 °C. To evaluate the relationship between pH value and time, a *t* test was conducted with a significance level of 0.05 (α = 0.05).

A mass uniformity test of the delivered dose from multidose containers, Eur. Ph. 2.9.27 [38], was conducted. Considering that we are dealing with suspensions, where the API is not completely dissolved in the medium, a content uniformity test could be used to calculate it to ensure that the right amount of API is in each dose [39]. The individual content of 10 doses could be used to calculate the acceptance value of each formulation. This would be more precise than the first test and could detect formulations with individual values within the limits ± 10% but with an AV higher than 15, the AV limit for 10 doses in the Eur. Ph. [40]. The samples were treated using the previously described extraction method (Section 2.6.1).

Viscosity measurements were conducted on the developed suspensions as this is a crucial parameter in avoiding API sedimentation and ensuring dosage homogeneity. A viscosimeter (LVDV-II Brookfield^®^, Essex, UK) was employed for this purpose. Viscosities were measured using different spindles, SC4-18 for L1-MC and SC4-25 for L2-XG, with sample volumes of 8 mL and 16 mL used, respectively. Following compounding at room temperature (25 ± 0.1 °C), measurements were taken under the different storage conditions during the stability test; stirring before removing the volumes is very important. There was a continual increase in the shear rate from 5 to 40 rpm over 45 min for L1-MC and from 80 to 200 rpm over 35 min for L2-XG. In both cases, the speed was increased by 5 rpm per 5 min. Data were processed with the Wingather^®^ 32 program Brookfield (Essex, UK). All measurements were performed in triplicate using a torque range of 10% to 90%.

#### 2.7.2. Quality Control for Solid Formulations

A digital calliper (SESA Tools, S.A., Gipuzkoa, Spain) was used to measure the dimensions of the orodispersible tablets and printlets.

As crucial quality parameters, weight variability, content uniformity, and dispersion fineness were assessed in accordance with Ph. Eur. guidelines [40,41]. The samples were treated using the previously described extraction method (Section 2.6.2). Although the pharmacopoeia does not contain recommendations for these dosage forms, these tests have been used as an approximation to evaluate the qualities of moulded tablets and printlets created for the oral route, with the primary focus on the quality evaluation of compressed tablets. The disintegration time of 6 moulded tablets and printlets for each batch was determined using a disintegration tester (Disintegration tester ZTx20, Erweka, Germany), according to EP 2.9.1. Tablets [42] must disintegrate in less than 3 min to be considered orodispersible dosage forms.

### 2.8. Chemical Stability

Chemical stability was evaluated following ICH recommendations [43]. To perform this test, liquid formulations were stored at 5.0 ± 0.1 °C (Refrigerator-stove P-selecta. Wedilow type), 25 ± 1 °C (ULP500, Memmert GmbH, Büchenbach, Germany), and 40.0 ± 0.1 °C (Climatic chamber ICH110L, Memmert GmbH, Büchenbach, Germany). In the case of solid formulations, moulded tablets and printlets were placed in a closed PVC blister package and stored at 25 ± 1.32 °C/60 ± 5% RH in a climatic chamber (Selecta Medilow climatic chamber, J.P. Selecta SA, Barcelona, Spain).

### 2.9. In Vitro Mucoadhesive Test

**Figure 1:**
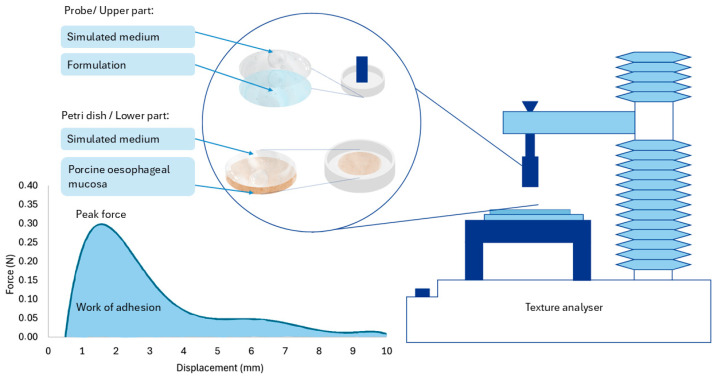
Schematic representation of the texture analyser. Illustration based on Amorós-Galicia et al., 2023 [45]. On the right-hand side of the figure, the texture analyser is shown. The upper probe holds the simulated fluid along with the formulation to be evaluated, while a Petri dish containing the oesophageal tissue covered with simulated medium is placed in the lower part. The graph represents a typical force–displacement curve obtained during the bioadhesion test.

The bioadhesion test was conducted using a texture analyser (TA.HDplusC, Stable Micro Systems Ltd., Surrey, UK). To simulate the absorption conditions, the lower part of the probe was covered with fresh porcine oesophageal mucosa, obtained from the municipal slaughterhouse of Tenerife (Santa Cruz de Tenerife, Spain). The mucosa was cleaned with normal saline solution (0.9% NaCl) [44] and cut into round pieces of a similar size (5 cm in diameter). These pieces were placed on Petri dishes and then coated with 1 mL of simulated medium, and a specified volume of each formulation was added. To enable a direct comparison of bioadhesion, the amount of each formulation was standardised to 1 mg of BUD. This corresponded to 5 mL of L1-MC (0.2 mg/mL BUD); 4 mL of L2-XG (0.25 mg/mL BUD); one M1 moulded tablet (1 mg of BUD); one P1 printlet (1 mg of BUD); and one P1-XG printlet (with the same composition as P0.5-XG but adjusted to 1 mg of BUD). The probe was then lowered to 0.1 mm/s until it came into contact with the formulation. At this point, it began to exert a force of 0.5 N for 60 s [45]. Subsequently, the upper part of the texture analyser was raised at a speed of 0.1 mm/s [46] while the formulation provided resistance to the traction. All formulations were assessed 3 times (n = 3). Figure 1 shows a schematic representation of the texture analyser.

The bioadhesion work, representing the energy required to detach the formulation from the mucosa, was calculated as the area under the curve (AUC) corresponding to the force exerted by the formulation during the upward movement of the probe. The results are expressed in terms of two parameters: peak force (the maximum force required for detachment) and the work of adhesion (the total energy required for detachment).

Statistical relevance was established at *p* < 0.05. The Excel Data Analysis ToolPak was used as a statistical tool. Data are expressed as mean ± SD, and statistical significance was measured using a one-way ANOVA.

## 3. Results and Discussion

### 3.1. Formulations Developed

Three batches of oral viscous suspensions (L1-MC and L2-XG) and moulded tablets were prepared, requiring 30 and 20 min per batch, respectively. Three batches of 100 printlets were elaborated automatically, in 1 h each.

The SSE-P sensor integrated into the printing platform enabled the characterisation of the physical properties of each of the semi-solid masses developed prior to printing the batches, which allowed for the determination of the semi-solid masses’ density from their extruded mass and the estimation of the final dimensions of printed objects required to deliver the target dose of BUD [34]. The results of the extrudability analysis are presented in Table 5.

By comparing the start flow pressure and the maximum applied pressure for each developed formulation, we could anticipate the influence of these parameters on the mass variation of the initial printlets [34]. The P0.5-XG semi-solid mass exhibited the highest-pressure value (113.310 kPa) due to the inclusion of XG, followed by P0.5 and P1.

**Figure 2:**
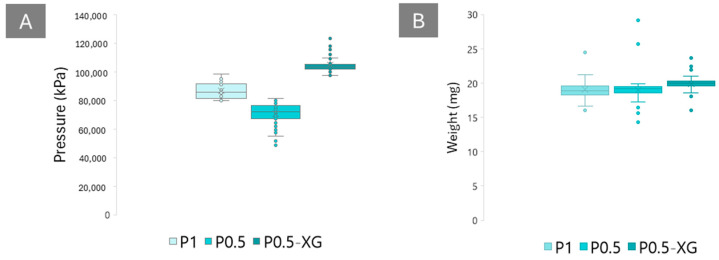
Boxplot with outliers of the pressure (**A**) and weight (**B**) of the printlets developed. • represents cases or rows with values greater than the height of the boxes multiplied by 1.5.

The results of the printability test are shown in Figure 2, with the most relevant parameters, such as the mean printing pressure during the process, highlighted in Figure 2A. As anticipated from the prior extrudability analysis, the P0.5-XG formulation exhibits the highest pressure values. The average weight of the dosage units for each formulation is shown in Figure 2B. The recorded weights of the printed units enable the formulator to identify and exclude dosage units that deviate from the obtained average weight, thus ensuring a consistent BUD content in each formulation.

The final dose unit’s quality can be safely assured due to the inclusion of the SSE-P sensor. This has effectively addressed the difficulty of creating dose units with an extremely low API content, such as with 0.5 mg of BUD (2.5%) and 1 mg of BUD (5%) [34,47]. This contrasts with the moulded tablets (M1), where the final dose depends on the accurate filling of the mould cavities; the addition of excess BUD is required to compensate for potential losses during the moulding process.

### 3.2. Extraction and Validation of Budesonide Method 

The ANOVA of the linear regression confirmed the linearity of the proposed method for the quantification of BUD through the rejection of the null hypothesis of deviation from linearity at a significance level of 0.05 (α = 0.05). The coefficient of variation of the method was 4.92%. The equation of the regression line is Area (μV·s^−1^) = 20,730 · C (μg/mL) −3241; r^2^ = 0.99. It is precise (0.54% n = 6), accurate (102.5% n = 9), and has detection and quantification limits of 1.34 and 4.06 μg/mL, respectively.

**Figure 3:**
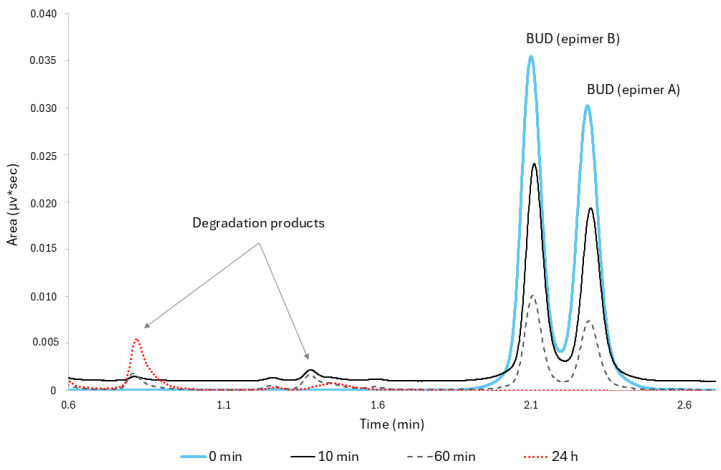
Overlaid UHPLC chromatograms of BUD subjected to stress conditions in basic medium (NaOH 0.1 M) at different times (0 min; 10 min; 60 min; and 24 h). Epimer B (22R); Epimer A (22S).

In addition, the detection of BUD degradation and observation of different products are demonstrated by the method shown in Figure 3.

The average of the extraction yields of BUD for the different methods used to treat the different samples were close to 100% of the BUD and expressed as a percentage of API: L1-MC 98.7 ± 0.2% (n = 10), L2-XG 98.7 ± 3.6% (n = 10), M1 103.6 ± 2.7% (n = 10), P1 99.7 ± 3.2% (n = 10), P0.5 104.9 ± 0.01% (n = 10), and P0.5-XG 103.9 ± 0.8% (n = 10). This shows that the excipients do not interfere with the API analysis and it is possible to extract the right amount of BUD from a complex matrix (methylcellulose, xanthan gum, glycerol, etc.). In addition, it is shown that the extraction method used for each formulation allows us to correctly extract BUD.

### 3.3. Liquid Formulations

Each liquid formulation had a transparent appearance when it was elaborated.

Table 6 shows the pH changes over time for the oral liquid formulations of BUD studied (L1-MC and L2-XG). There was no statistically significant difference between pH and time for all storage conditions (*p* > 0.05). The pH results support the chemical stability test, indicating that the BUD did not degrade. However, L2-XG, in contrast to L1-MC, directly incorporates the citrate proportion into its elaboration; this incorporation allows the formulator to prepare all the batches with the same initial pH, avoiding pH adjustment errors.

With no more than two individual masses deviating from the average mass by more than 10%, and none exceeding a 20% deviation, both formulations met the Ph. Eur. test for the mass uniformity of multidose containers, 2.9.27 [38]. Table 7 shows the results.

Both L1-MC and L2-XG met the dose uniformity test, with acceptance values (AV) of 4.76 and 5.97, respectively; both AVs were below the required limit of 15 (n = 10) [40]. The results are presented in Table 8.

**Figure 4:**
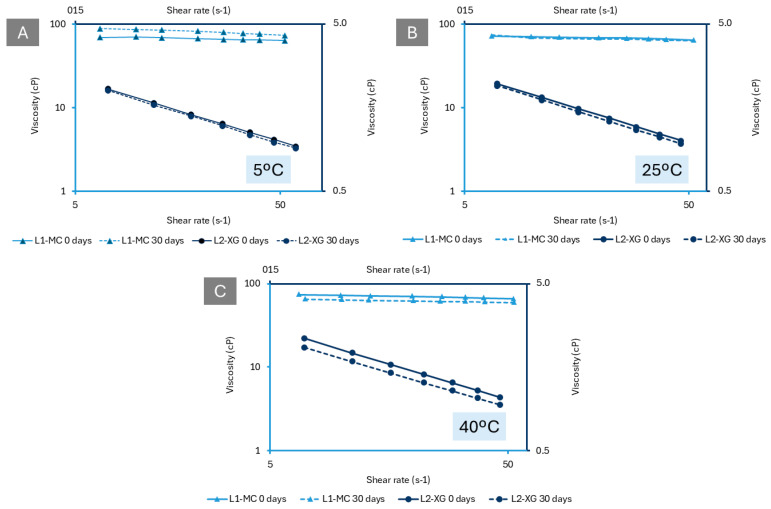
Plots of the log of viscosity versus the log of shear rates of L1-MC and L2-XG during stability tests under different storage conditions. (**A**). 5 °C; (**B**). 25 °C; and (**C**). 40 °C.

In terms of viscosity, for L1-MC, the data show a linear relationship between viscosity and shear rate, indicating Newtonian behaviour (Figure 4A–C). In Newtonian fluids, viscosity remains constant regardless of the shear rate. This means that the viscosity of L1-MC does not change when shaken or stirred. Observations reveal an increase in viscosity in the formulation stored at 5 °C over time (Figure 4A), while viscosity decreases in the formulation stored at 40 °C (Figure 4B); typical behaviour for Newtonian fluids [48,49].

In contrast, L2-XG exhibits non-Newtonian behaviour, specifically pseudoplastic behaviour, as its viscosity changes with the shear rate [48,50,51]; Figure 4A–C. This means that L2-XG’s viscosity decreases with vigorous shaking, facilitating the easier resuspension of the API and ensuring a more homogeneous formulation after standing. Notably, the viscosity of L2-XG decreases over time at all temperatures.

For enhanced efficacy, L2-XG’s pseudoplastic behaviour is advantageous because its higher flow resistance allows the suspension to adhere more effectively to the oesophageal mucosa. This ensures that BUD remains in contact with the mucosa for a longer period, maximising its therapeutic effect [52,53].

### 3.4. Solid Formulations

**Figure 5:**
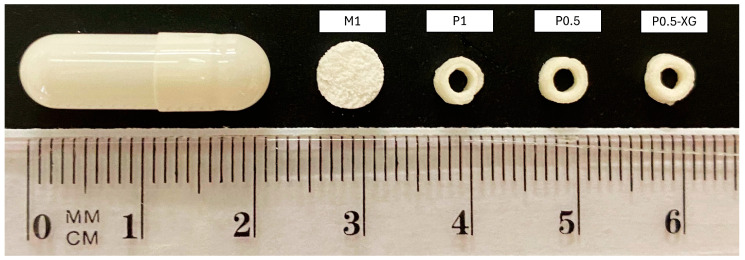
Visual comparison of the shape and size of the moulded tablets (M1) and printlets (P1, P0.5, P0.5-XG) with a size 0 capsule.

The different sizes of the solid formulations obtained are shown in Table 9 and the shape of each formulation is shown in Figure 5. As can be seen, the sizes of the solid formulations are suitable for paediatric administration in patients aged 2 years and above [21,23,54].

Table 10 shows the results of the mass uniformity test of single-dose preparations [41]. All solid dosage forms passed the mass uniformity test of single-dose preparations, with no individual mass deviating from the average mass by more than the allowed limits: 7.5% for M1 and 10% for the printlets P1, P0.5, and P0.5-XG.

The results obtained from the uniformity assay of the dosage unit, using the process described for the solid formulations, are shown in Table 11. As is evident from the data, the acceptance value (AV) is less than 15 (below the pharmacopoeia limit for 10 samples). Hence, all formulations comply with the European Pharmacopoeia test [40].

However, it is important to note that while all formulations meet the requirements, the moulded tablets (M1) exhibited a higher relative standard deviation for mass and API content compared to the printlets. This discrepancy can be attributed to the inherent variability in the manual filling process of the cavity plate for moulded tablets, which aligns with findings from previous studies [26]. In contrast, orodispersible printlets benefit from automated production, where a computerised system precisely controls the extrusion of the wet mass, ensuring greater consistency in each unit.

The disintegration times of all solid formulations were under 3 min, fulfilling the criteria for orodispersible tablets as defined by the European Pharmacopoeia [42].

### 3.5. Chemical Stability of Budesonide Formulations

The chemical stability results for liquid formulations can be seen in Table 12. The stability period established at 5 °C was 30 days for both oral viscous formulations L1-MC (102.56 ± 1.02% DV) and L2-XG (95.93 ± 4.17% DV), which remained at more than 90% of their declared value. However, at 25 and 40 °C, despite maintaining more than 90% of their declared value, their standard deviation increases. This increase in standard deviation raises concerns about the formulations’ content uniformity, homogeneity, and chemical stability, potentially compromising the quality, safety, and efficacy of the administered doses.

Table 13 shows the chemical stability results for the developed solid formulations. The established stability period at 25 °C was 30 days for all solid formulations, all of which retained more than 90% of their declared value at 30 days.

### 3.6. Mucoadhesive Properties

**Figure 6:**
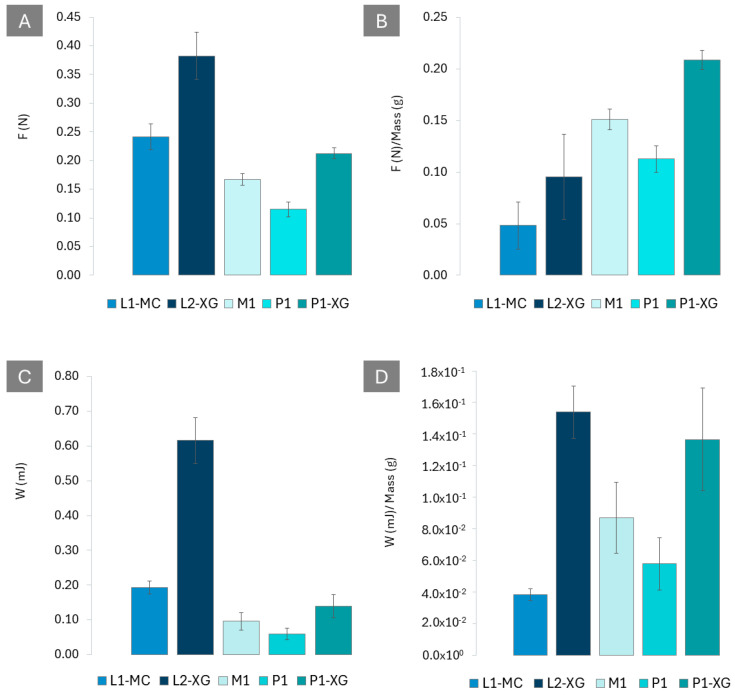
Comparative analysis of the maximum bioadhesion results for all formulations studied. (**A**). Average maximum bioadhesion forces for the doses of each formulation studied. (**B**). Average maximum bioadhesion forces normalized to 1 g of formulation. (**C**). Average work adhesion, W (mJ), for the doses of each formulation studied. (**D**). Average work adhesion, W (mJ), normalised to 1 g of formulation.

The peak force and work of the adhesion results from the in vitro mucoadhesive test are presented in Figure 6. Regarding mucoadhesive properties, when comparing all formulations at the same dose (1 mg of BUD), as shown in Figure 6A,C, the oral viscous suspension L2-XG exhibits the highest maximum bioadhesion peak force (0.38 ± 0.04 N, n = 3) and work of adhesion (0.62 ± 0.48 mJ, n = 3), followed by L1-MC (0.24 ± 0.02 N and 0.19 ± 0.02 mJ, n = 3); P1-XG (0.21 ± 0.01 N and 0.14 ± 0.03 mJ n = 3); and M1 (0.17 ± 0.02 N and 0.10 ± 0.03 mJ n = 3), while, finally, P1 demonstrates the lowest values in both parameters: peak force (0.113 ± 0.012 N, n = 3) and work of adhesion (0.058 ± 0.017 mJ, n = 3). This difference is primarily attributed to the variation in the surface area of the oesophageal mucosa that each formulation can cover.

In the comparison within formulation types, L2-XG exhibited significantly higher in vitro mucoadhesion than L1-MC, with a 1.6-fold increase in peak force and a 3.2-fold increase in work of adhesion. Among the solid formulations, P1-XG demonstrated the highest mucoadhesion, surpassing both M1 and P1 in terms of peak force and work of adhesion.

To assess their intrinsic in vitro mucoadhesive properties, independent of dose or volume, the maximum mucoadhesion peak force and work of adhesion values were normalised to 1 g for each formulation, Figure 6B,D. This analysis reveals that P1-XG exhibits the highest mucoadhesivity in terms of peak force (Figure 6B), while L2-XG maintains its superior position in terms of work of adhesion (Figure 6D). The remaining formulations follow in decreasing order, as shown in Figure 6B,D.

The in vitro mucoadhesion test characterises the mucoadhesive properties of the developed formulations, offering valuable insights into their interaction with the oesophageal mucosa. The results suggest the potential for prolonged residence time, aligning with previous reports [55,56]. Notably, the presence of XG emerges as a key factor in enhancing bioadhesion for both viscous liquid (L2-XG) and solid formulations (P1-XG). This effect is attributed to the presence of -COO- groups, corroborating earlier research [3].

The observed impact of formulation type and surface area on mucoadhesion measurements highlights the necessity of developing standardised methodologies that account for these variables.

## 4. Conclusions

A validated analytical method was developed, enabling the accurate quantification of BUD, alongside an efficient extraction method for recovering low doses of BUD from complex matrices, including viscous suspensions, moulded tablets, and 3D-printed dosage forms. All developed formulations comply with Ph. Eur. quality control standards and demonstrate chemical stability for 30 days (at 5 °C for liquid formulations and 25 °C for solid formulations).

The liquid formulation with xanthan gum (L2-XG) and the printlets containing xanthan gum (P1-0.5-XG) exhibited superior mucoadhesive properties, suggesting their potential for enhanced drug retention and improved therapeutic outcomes. L2-XG exhibited significantly higher in vitro mucoadhesive properties than L1-MC. Among the solid formulations, P1-XG demonstrated the highest mucoadhesive properties, surpassing both P1 and M1 in terms of peak force and work of adhesion.

This study is the first to develop orodispersible budesonide printlets at very low doses (1 mg and 0.5 mg) specifically for paediatric use, demonstrating the feasibility of 3D printing via semi-solid extrusion for producing accurate and patient-friendly dosage forms. The use of the SSE-P sensor for process control further enhances this promising approach to manufacturing low-dose paediatric formulations. Three-dimensional printing’s potential to create individualised dosage forms positions it as a revolutionary tool for paediatric drug delivery, particularly in the treatment of conditions like EoE.

## Figures and Tables

**Table 1:** Attributes necessary to ensure the quality target product profile of the developed formulations.

Attribute	Development Target		
Route of administration	Oral		
Dose strength	1 mg	1 mg	0.5 mg
Dosage form	Viscous oral liquid formulation	Orodispersible solid dosage form	
Dosing regimen	Once/twice daily		
Impurities	Degradation products		
Requirements: to ensure patient safety and efficacy throughout shelf life	All appropriate quality criteria: identification; appearance; mass uniformity; content uniformity; chemical stability; mucoadhesive properties.	All appropriate quality criteria: identification; appearance; disintegration; mass uniformity; content uniformity; chemical stability; mucoadhesive properties.	
Shelf life	Minimum 30 days		

**Table 2:** Composition of oral viscous formulations (% *w*/*v*).

Composition	L1-MC	L2-XG
Budesonide	0.02	0.025
Glycerine	2.52	12.61
Methylcellulose	1.00	-
Saccharin sodium	0.05	0.08
Nipagin sodium	0.05	-
Nipasol sodium	0.02	-
Sodium citrate	0.05	-
Citric acid monohydrate	0.10	-
Xanthan gum	-	2.00
Sodium benzoate	-	0.19
EDTA	-	0.10
Water, Ultrapure	96.19	85.00
Total	100.00	100.00

**Table 3:** Composition of solid formulations, moulded tablets (M1), and printlets (P1; P0.5; P0.5-XG) (% *w*/*w*).

	Moulded Tablets	Printlets		
Composition	M1	P1	P0.5	P0.5-XG
BUD	0.69 ^1^	1.74	0.83	0.87
Lactose, monohydrate	60.75	14.78	14.66	14.67
Ac-Di-Sol^®^	-	10.43	10.33	10.78
Sucrose	26.23	-	-	-
PVP ^2^	0.59	3.48	3.33	3.48
Xanthan Gum	-	-	-	0.63
Ethanol	2.78	16.47	19.99	16.47
Apple essence	0.36	1.33	1.28	1.33
Water, Ultrapure	8.53	51.77	49.58	51.77
Total	100.00	100.00	100.00	100.00

^1^ M1 requires an additional 40% BUD to compensate for compounding losses due to the moulding process, which depends on the accurate filling of the cavity plates [26,27]. ^2^ PVP: polyvinylpyrrolidone.

**Table 4:** Compression/shrinkage cycle. Nozzle inner diameter of 0.61 mm, syringe diameter of 20.20 ga, and extrusion temperature of 40 °C.

Step	Action	Plunger Speed (mm/s)	Distance (mm)	Time (s)
1	Downward force on plunger cartridge	0.005	5	1000
2	Hold time	0	0	60
3	Retraction of the same distance	0.050	5	100
4	Final wait time	0	0	60

**Table 5:** Results of extrudability analysis of each semi-solid mass developed (P1; P0.5; P0.5-XG).

Semi-Solid Masses	P1	P0.5	P0.5-XG
Extrudability speed (mm/s) *	0.010	0.010	0.010
Start flow pressure (yield point) (kPa)	38.004	62.604	61.051
Maximum applied pressure (kPa)	59.716	66.879	113.310
Steady flow pressure (kPa)	56.508	62.604	105.649
Flow cessation pressure (kPa)	39.111	49.188	82.193
Recoverable stress (%)	94.66	93.61	93.23

* This extrusion speed was estimated for a printing speed of 10 mm/s, taking into account the syringe diameter (20.20 mm) and the nozzle diameter (0.6 mm) used.

**Table 6:** pH at preparation time (t = 0) and at the end of different storage conditions (t = 30 days) of L1-MC and L2-XG.

Oral Viscous Formulations	Conditions	pH_0_–pH_30_
L1-MC	5 °C	4.15 ± 0.01–4.18 ± 0.01
	25 °C	4.09 ± 0.01–4.28 ± 0.03
	40 °C	4.12 ± 0.01–4.30 ± 0.08
L2-XG	5 °C	6.04 ± 0.00–5.97 ± 0.01
	25 °C	5.94 ± 0.01–5.95 ± 0.01
	40 °C	5.91 ± 0.01–5.81 ± 0.09

**Table 7:** Results for the mass uniformity of multidose containers. Evaluation at 1 mg BUD dose: 5 mL of L1-MC [0.2 mg/mL] and 4 mL of L2-XG [0.25 mg/mL].

	L1-MC		L2-XG	
Average (mg)	5.084		3.771	
SD	0.029		0.143	
Limits	LL	UL	LL	UL
±10%	4.576	5.593	3.394	4.148
±20%	4.068	6.101	3.017	4.525

SD: standard deviation; LL: lower limit; UL: upper limit.

**Table 8:** Results of the uniformity content test of liquid formulations, expressed as % of DV.

	L1-MC	L2-XG
Average (%DV)	97.22	102.93
SD	1.45	4.33
RSD	1.49	4.21
AV	4.76	5.97

DV: declared value; SD: standard deviation; RSD: relative standard deviation; AV: acceptance value.

**Table 9:** Dimensions of solid formulations.

Parameters	Moulded Tablets	Printlets		
	M1	P1	P0.5	P0.5-XG
Diameter (n = 10)	5.91 ± 0.08 mm	4.12 ± 0.08 mm	4.16 ± 0.08 mm	3.99 ± 0.16 mm
Thickness (n = 10)	3.27 ± 0.10 mm	2.08 ± 0.09 mm	2.17 ± 0.10 mm	2.01 ± 0.14 mm

**Table 10:** Results of the mass uniformity test of single-dose preparations.

	M1		P1		P0.5		P0.5-XG	
Average (mg)	104.85		19.57		18.46		19.88	
SD	5.71		0.44		0.48		0.56	
RSD	5.45		2.23		2.59		2.83	
Limits	LL	UP	LL	UL	LL	UL	LL	UL
±10%	-	-	17.61	21.53	16.61	20.30	17.90	21.87
±7.5%	96.99	112.72	-	-	-	-	-	-

SD: standard deviation; RSD: relative standard deviation; LL: lower limit; UL: upper limit.

**Table 11:** Results of the uniformity content test of solid formulations, expressed as % of DV.

	M1	P1	P0.5	P0.5-XG
Average (% DV)	101.59	101.76	103.45	106.00
SD	5.38	2.65	3.17	1.79
RSD	5.30	2.60	3.08	1.69
AV	12.96	6.36	8.70	4.54

DV: declared value; SD: standard deviation; RSD: relative standard deviation; AV: acceptance value.

**Table 12:** Results of the stability test of liquid formulations, expressed as mean ± standard deviation (n = 3) of a % of the declared value.

Oral Viscous Formulations	Storage Conditions	0 Days	15 Days	30 Days
L1-MC	5 °C	100.00	100.59 ± 3.16	102.56 ± 1.02
	25 °C	100.00	101.26 ± 8.87	98.08 ± 5.21
	40 °C	100.00	99.00 ± 4.39	97.92 ± 6.25
L2-XG	5 °C	100.00	103.97 ± 3.47	95.93 ± 4.17
	25 ° C	100.00	102.41 ± 0.82	101.36 ± 8.23
	40 °C	100.00	101.37 ± 3.89	97.76 ± 10.77

**Table 13:** Results of the stability test of solid formulations, expressed as mean ± standard deviation (n = 3) of a % of the declared value.

Moulded Tablets and Printlets	Storage Conditions	0 Days	30 Days
M1	25 °C	100.00	100.82 ± 3.34
P1		100.00	97.56 ± 9.39
P0.5		100.00	102.78 ± 2.04
P0.5-XG		100.00	99.88 ± 1.34

## Data Availability

The data can be shared up on request.

## Abbreviations

3D	Three-dimensional
API	Active pharmaceutical ingredient
AUC	Area under the curve
BUD	Budesonide
EMA	European Medicines Agency
EoE	Eosinophilic oesophagitis
EP	European Pharmacopoeia
F	Force
FP	Fluticasone propionate
ICH	International Conference of Harmonisation
MC	Methylcellulose
OVB	Oral viscous budesonide
Printlets	3D-printed dosage forms
QTTP	Quality target product profile
SSE	Semi-solid extrusion
STC	Swallowed topical corticosteroids
W	Work
XG	Xanthan gum
